# Pharmacokinetics of β-Alanine Using Different Dosing Strategies

**DOI:** 10.3389/fnut.2018.00070

**Published:** 2018-08-17

**Authors:** Jan Stautemas, Inge Everaert, Filip B. D. Lefevere, Wim Derave

**Affiliations:** Department of Movement & Sports Sciences, Ghent University, Ghent, Belgium

**Keywords:** β-alanine, sports supplements, pharmacokinetics, personalized nutrition, carnosine

## Abstract

**Introduction:** The ergogenic response following long-term ingestion of β-alanine shows a high inter-individual variation. It is hypothesized that this variation is partially caused by a variable pharmacokinetic response induced by inferior dosing strategies. At this point most supplements are either taken in a fixed amount (× g), as is the case with β-alanine, or relative to body weight (× g per kg BW), but there is currently neither consensus nor a scientific rationale on why these or other dosing strategies should be used. The aim of this study is to objectify and understand the variation in plasma pharmacokinetics of a single oral β-alanine dose supplemented as either a fixed or a weight-relative dose (WRD) in an anthropometric diverse sample.

**Methods:** An anthropometric diverse sample ingested a fixed dose (1,400 mg) (*n* = 28) and a WRD of β-alanine (10 mg/kg BW) (*n* = 34) on separate occasions. Blood samples were taken before and at nine time points (up to 4 h) after β-alanine ingestion in order to establish a pharmacokinetic profile. Incremental area under the curve (iAUC) was calculated by the trapezoidal rule. Plasma β-alanine was quantified using HPLC-fluorescence.

**Results:** The variation coefficient (CV%) of the iAUC was 35.0% following ingestion of 1,400 mg β-alanine. Body weight explained 30.1% of the variance and was negatively correlated to iAUC (*r* = −0.549; *p* = 0.003). Interestingly, the CV% did not decrease with WRD (33.2%) and body weight was positively correlated to iAUC in response to the WRD (*r* = 0.488; *p* = 0.003).

**Conclusion:** Both dosing strategies evoked an equally high inter-individual variability in pharmacokinetic plasma profile. Strikingly, while body weight explained a relevant part of the variation observed following a fixed dose, correction for body weight did not improve the homogeneity in β-alanine plasma response. We suggest to put more effort into the optimization of easy applicable and scientifically justified personalized dosing strategies.

## Introduction

In sports nutrition, there is no uniformity in dosing strategies of ergogenic supplements to adults. A fixed dose (FD; × g per person per day) is by far the easiest to implement in daily practice and allows straightforward packaging and marketing. On the other hand, dosing is sometimes normalized for anthropometric characteristics. The weight-relative dose (WRD; × g per kg body weight per day), which is by far the most popular normalizing strategy, is somewhat less practical to prescribe but may seem more adequate. The WRD is expected to correct for differences in body size and weight, which can easily differ by a factor 3 between, say, a female gymnast and a male American Football lineman. There is a striking paucity in the nutrition literature in general and in the sports nutrition literature in particular, relating to the pros and cons of these two, or any other normalization approach and even more so relating to the physiological differences and direct comparison of different approaches.

β-alanine (BA) is classified as a Group A supplement in the Sport Supplement Program of the Australian Institute of Sport^1^ as it has been shown to improve performance of exercises lasting between 0.5 and 10 min (1). Its ergogenic effect is achieved by the intra-muscular increase of the dipeptide carnosine that is constituted from histidine and its rate-limiting precursor BA. Carnosine has multiple biochemical properties whereof its ability to buffer protons most likely is the major determinant that explains its ergogenic potential (2).

Currently, a FD is the most popular strategy in BA supplementation although different absolute doses are used (1). In the first report of BA as a human food supplement, Harris et al. investigated the pharmacokinetics of three different BA doses supplemented as WRD (10, 20, and 40 mg/kg BW) (3). Subsequently, these authors switched to FD and investigated the effect of a 15-d supplementation protocol of 3.2 and 6.4 g/d (FD) on muscle carnosine. In follow-up, Hill et al. (4) performed the first study on BA as an ergogenic food supplement with a FD (4–6.4 g/day). Thereafter (almost) all research, by this and other groups, was performed with absolute dosing strategies, but without further rationale.

As in many other supplementation studies, the improvement in exercise performance following BA ingestion is characterized by a lot of variation. For example, Saunders et al. (5) found that performance changes ranged from +0.0 to +72.7% (CV% > 60%) in distance covered in a YoYo intermittent recovery test level 2 (5). This is not surprising, as many others have shown that the variation in muscle carnosine loading is quite high as well. For example, Blancquaert et al. (6) found a change of gastrocnemius carnosine from 3.3% up to 119.0% (CV% = 74%) in people receiving 6.4 g BA for 23 days (6). As Hill et al. (4) showed that the change in muscle carnosine explains part of the change in performance, we can hypothesize that by reducing the variation in muscle carnosine increments, the ergogenic effect might also be more uniform. Interestingly, the coefficient of variation in BA's pharmacokinetic response was reported to be 32% in a standardized setting (7). As it was shown that muscle carnosine loading was correlated to plasma BA concentration in mice (8) and to fasted plasma BA concentrations after a supplementation period in humans [unpublished, data of Blancquaert et al. (6)], it is very likely that the variation in carnosine loading and therefore ergogenic response is explained, in part, by the variation in the acute pharmacokinetic profile of BA.

As it is the objective of any sport supplement to optimize performance, it is obvious that athletes require correct and perhaps personalized dosing that induces a homogenous pharmacokinetic and ergogenic response. Providing a subtherapeutic dose might fail to affect performance, whereas supratherapeutic dosing might decrease performance or could even cause (unhealthy) side effects. In case of BA, low ingestion leads to the lack or low increases of carnosine, most likely insufficient to affect performance (4, 9). On the other hand, a supratherapeutic dose of BA can acutely cause discomfort in the form of paraesthesia (3). Due to the acute side-effects of BA, a slow release formula was developed in order to reduce peak concentration but maintain total pharmacokinetic response (AUC) (7). Some have also suggested that chronic ingestion of high doses of BA can cause a decline in muscle and plasma histidine (6, 10), whereby a negative effect on health or performance cannot be excluded. On the other hand, others did not observe a decline in muscle histidine following chronic BA supplementation (3, 11). In general, one could expect that with a FD the smaller/lighter people will be more prone to overdosing and the larger/heavier people to underdosing. However, it might equally be that with WRD the reverse is occurring (smaller people get underdosed and larger people overdosed). The latter was suggested for carbohydrate ingestion and bioavailability during exercise since it was argued that this is more dependent on GI tract characteristics rather than on body weight thereby pleading in favor of a FD for carbohydrates (12).

Summarized, there is at this point no scientific rationale to use a FD in BA supplementation. On the other hand, there is no information why other dosing strategies should be used. Based on the current knowledge there is no way to know which dosing strategy evokes the most homogenous plasma response. Therefore, the aim of this study is to objectify and to understand the variation in plasma pharmacokinetics of a single oral BA dose (FD or WRD) in an anthropometric diverse sample.

## Methods

### Subjects

Thirty-four subjects (age 25.1 ± 4.29 y; body weight: 70.4 ± 14.9 kg; height: 1.73 ± 0.11 m); volunteered to participate in the first part of this study (WRD) whereof 28 (age: 24.8 ± 4.25 y; body weight: 68.1 ± 15.2 kg; height: 1.72 ± 0.11 m) completed the second part (FD) (Table 1). All subjects were in good health and none of the participants was vegetarian. Both males and females (WRD: 19 males/15 females and FD: 14 males/females) from a mixed ethnic background were recruited. The study protocol was approved by the local ethical committee (Ghent University Hospital, Belgium) and written informed consent was obtained from all participants before the study.

**Table 1:** Anthropometric characteristics sample.

		**FD (1,400 mg)**	**WRD (10 mg kg^−1^)**
*N*	Men	14	19
	Women	14	15
Age (year)	Min	18	18
	Max	34	35
	Mean	24.8	25.1
	SD	4.3	4.3
Body weight (kg)	Min	46.0	46.0
	Max	104.5	104.5
	Mean	68.1	70.4
	SD	15.2	14.9
Height (m)	Min	1.50	1.50
	Max	1.94	1.94
	Mean	1.72	1.73
	SD	0.11	0.11

*FD, fixed dose; WRD, weight-relative dose*.

### Study design and sample collection

Subjects arrived fasted at the lab at two separate occasions. At arrival, a catheter was inserted in an antecubital vein and the first blood sample was taken (heparin tube). Hereafter, a standardized breakfast, consisting of white bread, hazelnut paste (Nutella) and semi skimmed milk, was consumed. The total calorie count of the breakfast was 23% (388 ± 50 kcal) of the resting metabolic rate (RMR) as calculated by the formula of Mifflin (13) (1,672 ± 204 kcal). Ten minutes after the start of the breakfast, a single dose of pure BA (Indis nv, Belgium) in gelules was ingested with water. Consecutively nine blood samples were taken after 20, 40, 60, 90, 120, 150, 180, 210, and 240 min. During the experiment, subjects drank water *ad libitum* and they refrained from any physical activity. All participants received a relative dose of 10 mg/kg body weight pure BA on the first test day, whereas 28 of the 34 participants returned for a second experimental test day where they received an absolute dose of 1,400 mg. To obtain information about the subjective feelings and the location of paraesthesia, the subjects received a standardized questionnaire at the end of every experimental day asking for the occurrence, intensity, localization, timing and description of possible discomfort/side-effects (7).

The absolute dose of 1,400 mg corresponded to a relative dose of 20.79 ± 4.45 mg/kg BW (range: 13.40–30.43 mg/kg BW), whereas the relative dose matched 703.5 ± 149.4 mg (range: 460–1,045 mg). Standardized allocation of experimental test days was used in order to test for potential paraesthesia occurring with the lower dose (WRD), before exposing subjects to a higher dose (FD).

### Determination of plasma metabolites by high-performance liquid chromatography

Heparin plasma samples were analyzed for BA using a previously published HPLC method (6). In short, heparin plasma samples were deproteinized using a 1:9 ratio of 35% sulfosalicylic acid. Plasma supernatant was mixed with ACCQ Fluor Borate buffer and Fluor reagent from the AccQtag Chemistry kit (Waters sa-nv, Belgium) in a 1:7:2 ratio. Standard solutions of BA were treated similarly before HPLC analyses. The derivatized samples were applied to a Waters Alliance HPLC system with the following parameters: XBridge BEH column (4.6 × 150 mm, 2.5 μm; Waters) heated to 37°C; fluorescence detector (excitation/emission wavelength: 250/395 nm); using a flow gradient containing different amounts of buffer A (10% eluent A [Waters], 90% ddH2O), buffer B (100% acetonitrile), and buffer C (100% ddH2O) at a flow rate of 1 mL.min^−1^.

### Pharmacokinetics and statistical analysis

Pharmacokinetics was investigated using a first order kinetic and non-compartmental model. Incremental area under the curve (iAUC) was calculated by subtracting the baseline of the AUC calculated using the trapezoidal rule. Cmax was determined as the maximal concentration measured, whereas Tm was determined as the time Cmax was reached. T_1/2_ was calculated as 0.693 divided by the elimination constant (k_e_), whereas the k_e_ was computed as −2.303 multiplied by the slope of the individual linear curve of the log10 from Cmax till the concentration at time 240 min (14). CV% (standard deviation divided by mean) was calculated for all variables. Before statistical analysis, normality of the continuous variables was checked using Shapiro-Wilk. Multivariate analyses of variance (MANOVA) was performed to determine the role of sex on the different pharmacokinetic parameters following FD. Pearson correlations and linear regression were performed between the anthropometric and pharmacokinetic parameters. All statistical analyses were performed using the Statistical Package for the Social Sciences (version 25.0; SPSS, Chicago, IL). Values are presented as mean ± SD and significance was assumed at *P* ≤ 0.05.

## Results

### Variability with FD

**Figure 1:**
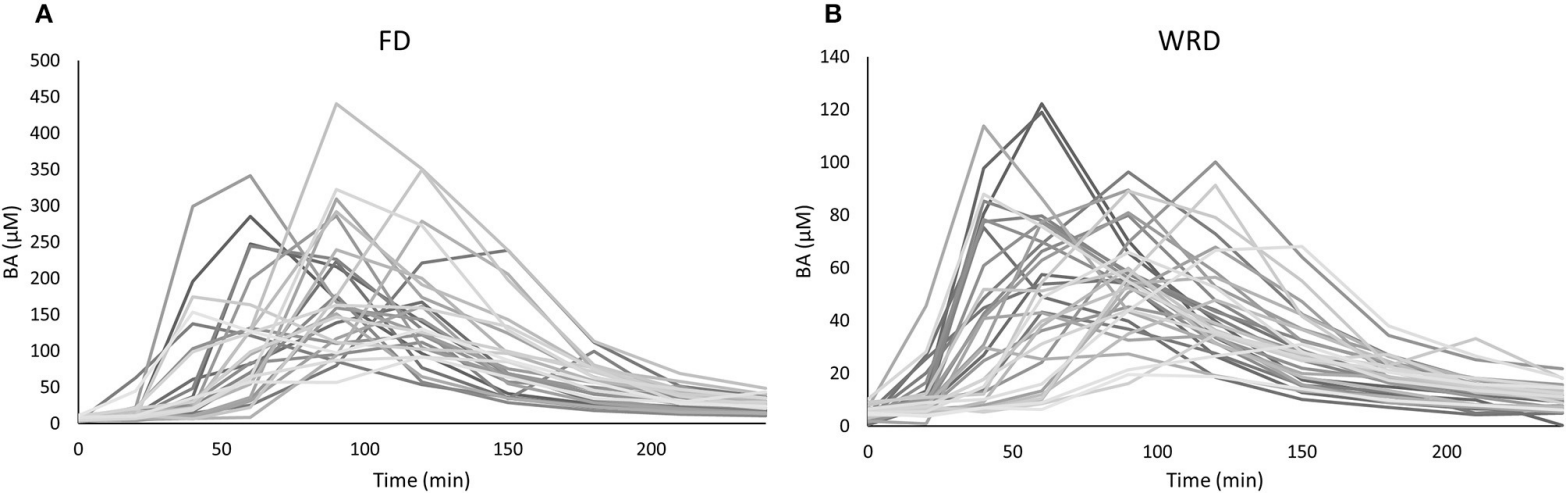
Variability in plasma pharmacokinetics in an anthropometric diverse sample following a **(A)** fixed (FD) and **(B)** weight-relative dose (WRD). Plasma β-alanine (BA) concentrations (μM) were determined before and at nine time points after the ingestion of 1,400 mg **(A)** or 10 mg/kg BW **(B)** BA. The observed variation in iAUC is 35.0 and 33.2%, respectively.

The inter-individual variability in iAUC and Cmax that could be observed in BA plasma pharmacokinetics following ingestion of a single FD of 1,400 mg BA was 35.0% (18,550 ± 6,495 μM.min; range: 9,334–38,183) and 40.2% (218.4 ± 87.9 μM; range: 91.7–440.5), respectively (Figure 1A) (Table 2). The T_1/2_ varied from 32.6 to 97.8 min and Tm varied from 40 to 150 min. These pharmacokinetic parameters were not significantly different between sexes (data not shown).

**Table 2:** Observed variation in iAUC and Cmax in both dosing strategies.

		**FD**	**WRD**
iAUC	%CV	35.0	33.2
	Fold difference (highest vs. lowest)	4.09	7.16
Cmax	%CV	40.2	37.5
	Fold difference (highest vs. lowest)	4.81	6.25

*FD, fixed dose; WRD, weight-relative dose*.

### Anthropometric factors underlying the variation with FD

**Figure 2:**
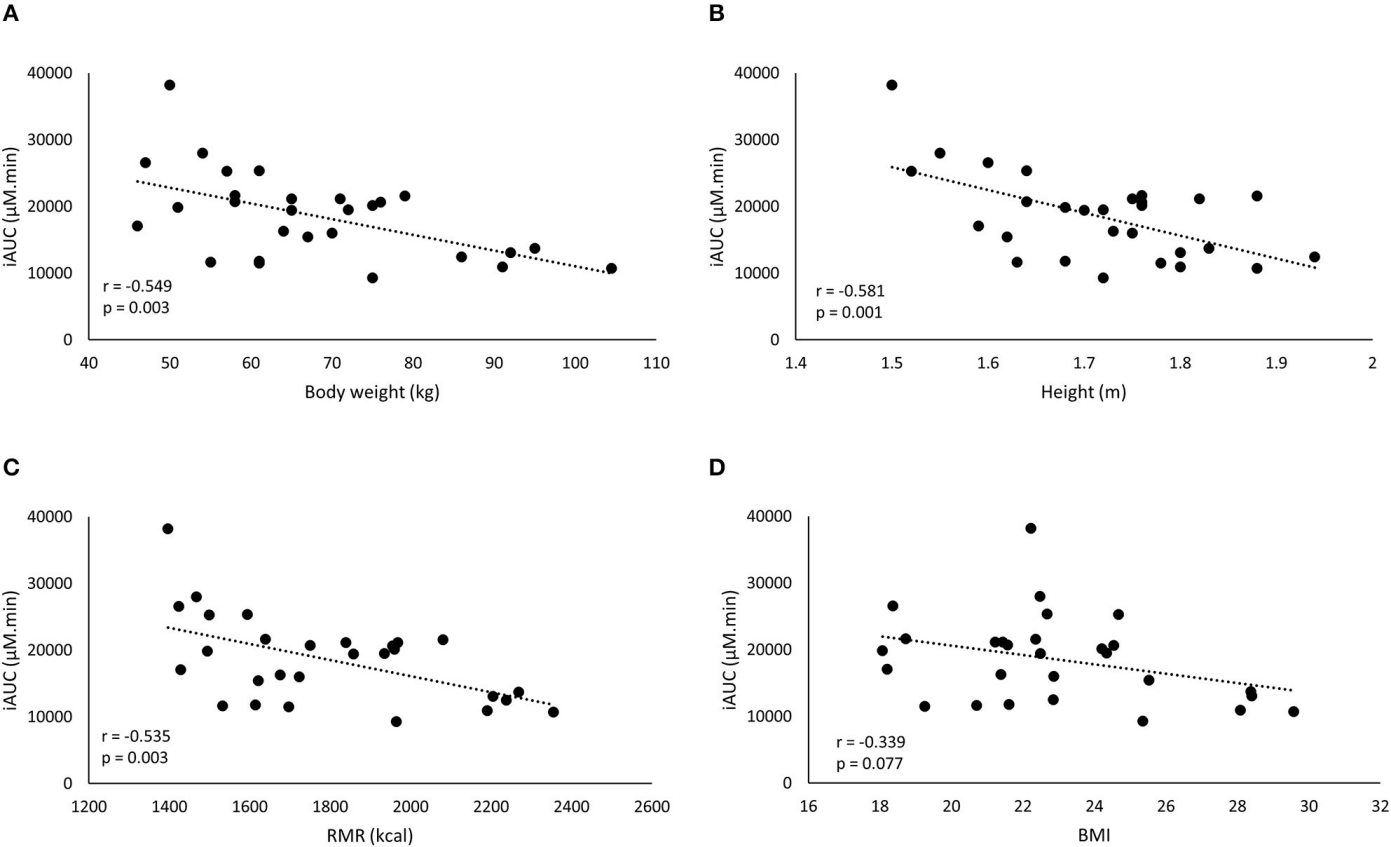
A negative correlation is observed between iAUC of the fixed dose and the anthropometric characteristics: body weight (BW) **(A)**, height **(B)**, resting metabolic rate (RMR) **(C)**, and BMI **(D)**.

The iAUC of the FD was negatively correlated with body weight, height and RMR, but not to BMI (Figure 2). The strongest correlation with plasma iAUC was found for height, explaining 33.8% of the variance, which was only slightly higher than the 30.1% explained by body weight (Table 3). Cmax of FD was also correlated to all anthropometrics (Table 3). Body weight had the highest explained variance (29.1%) for Cmax.

**Table 3:** Correlations between anthropometrics vs. iAUC and Cmax.

		**FD**		**WRD**	
		**iAUC**	**Cmax**	**iAUC**	**Cmax**
BW	*r*	−0.549^*^	−0.540^*^	0.488^*^	0.360^*^
	*r*^2^	0.301	0.291	0.238	0.130
Height	*r*	−0.581^*^	−0.531^*^	0.415^*^	0.313^$^
	*r*^2^	0.338	0.282	0.172	0.098
BMI	*r*	−0.339^$^	−0.378^*^	0.401^*^	0.289^$^
	*r*^2^	0.115	0.143	0.161	0.083
RMR	*r*	−0.535^*^	−0.522^*^	0.479^*^	0.364^*^
	*r*^2^	0.287	0.273	0.230	0.132

FD, fixed dose; WRD, weight-relative dose

* *p < 0.05*;

$ *0.05 < p < 0.10*.

### Variability with WRD

We also investigated the pharmacokinetic response following ingestion of 10 mg/kg BW BA (WRD). Despite this body weight correction of dosing, a nearly equally high inter-individual variability was observed (Table 2; Figure 1B) and the fold-difference between the highest and lowest responder was even more pronounced. The CV% of iAUC and Cmax was 33.2% (6,134 ± 2,038 μM.min; range: 1,424–10,201) and 37.5% (69.0 ± 25.9 μM; 19.6–122.2 μM), respectively.

**Figure 3:**
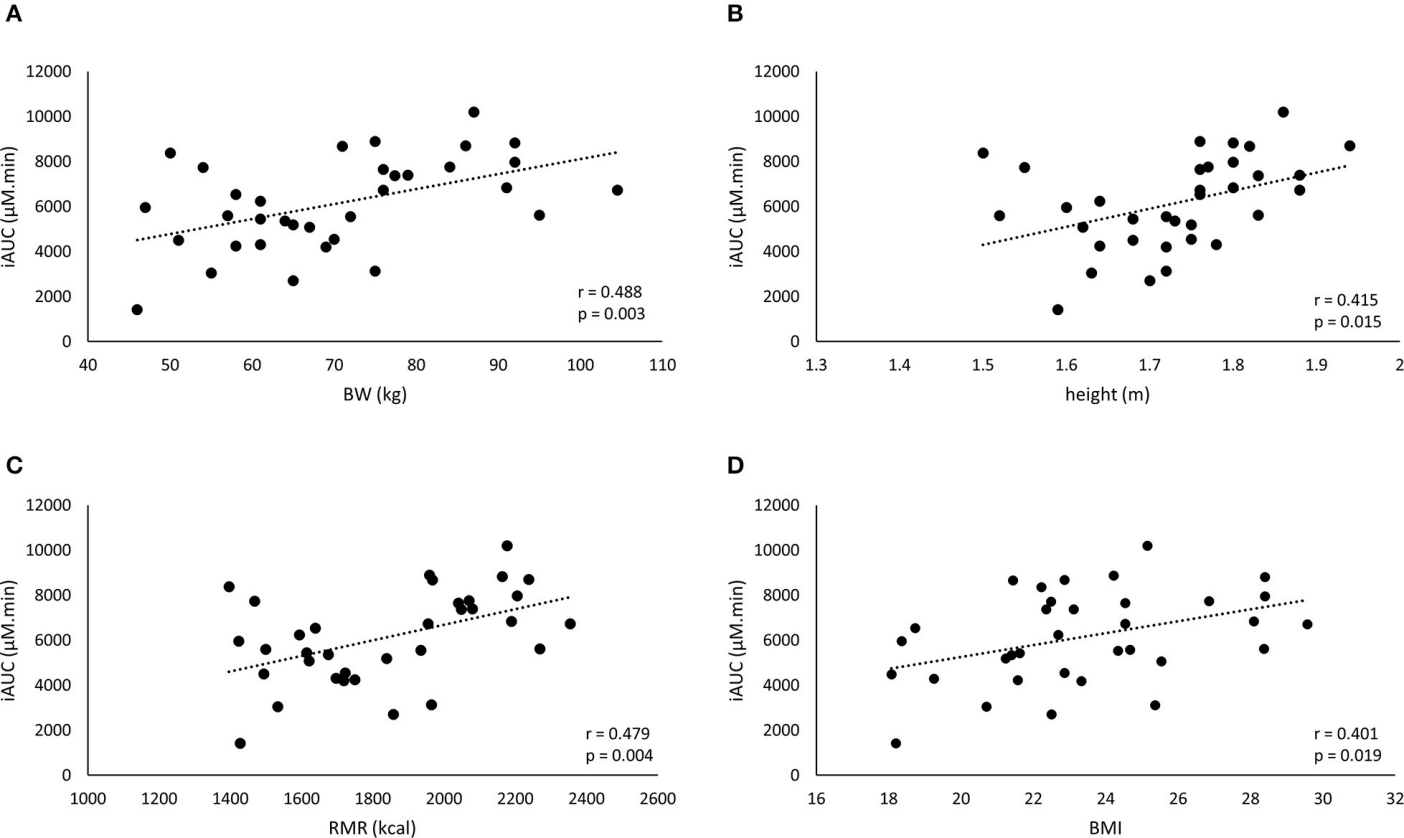
A positive correlation is found between the iAUC of the weight-relative dose and the anthropometric characteristics: body weight (BW) **(A)**, height **(B)**, resting metabolic rate (RMR) **(C)**, and BMI **(D)**.

The iAUC of the WRD was positively correlated to body weight, height, BMI, and RMR (Figure 3), while Cmax was correlated to body weight and RMR but not to height and BMI (Table 3).

### Relationship dose and iAUC

**Figure 4:**
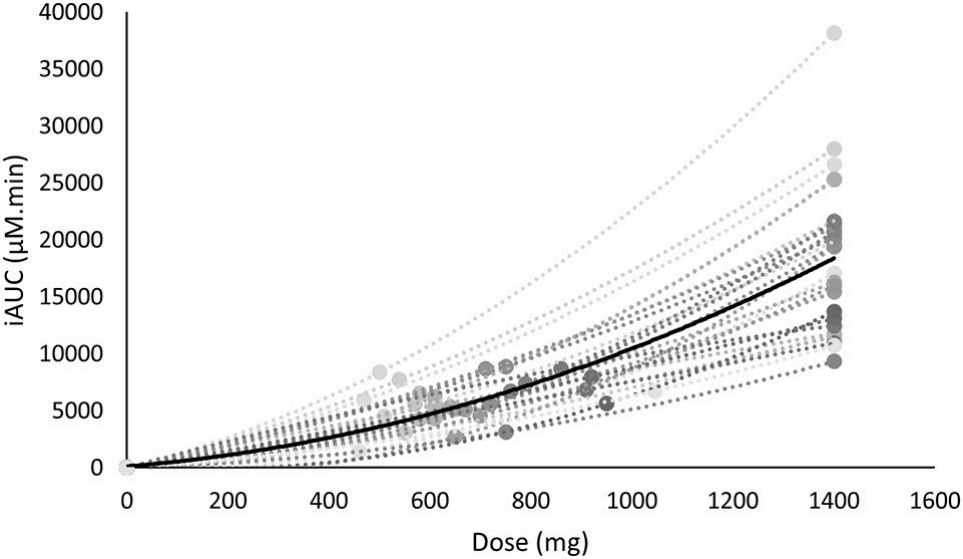
The iAUC of β-alanine is non-linearly related with the supplemented dose. The mean is shown in black, whereas the individual values are shown in gray.

As the FD and WRD were in a different absolute dose range, we investigated the link between dose and iAUC. There was a non-linear relationship between the dose and the iAUC on both the individual and the population level (Figure 4).

### Side effects

Nobody reported paraesthesia following WRD and two subjects reported paraesthesia following ingestion of FD. These subjects did not have the highest values of AUC, Cmax, Tm, or T_1/2_ of the evaluated population, but the moment of paraesthesia matched their individual Cmax value.

## Discussion

In this study, the inter-individual differences in pharmacokinetic response (iAUC) following BA ingestion are described. The iAUC is a crucial parameter because it reflects the concentration and duration of elevated plasma BA. It is assumed that the variation in acute BA pharmacokinetic profile reflects the variable response on carnosine loading and thereby ergogenic outcome following long-term BA supplementation. The coefficient of variation of the iAUC was 35.0% following a FD (Figure 1A), with a marked 4-fold difference between the lowest and highest iAUC. This variation resembles the 32% variation reported by Décombaz et al. (7). Seeking for an explanation for the high diversity in physiological response, it was observed that body weight explained a relevant part (30.1%) of the variability in plasma kinetic response. The negative correlation to body weight (*r* = −0.549; *p* = 0.003) signifies that heavy people received too little and light people received too much BA, to cause a homogenous response. The current observations call for a body weight correction to personalize dosing.

The striking observation of the current study is that the body weight correction (10 mg/kg BW) did not improve homogeneity in BA plasma response, with a CV% of 33.2% and an even more pronounced 7-fold difference between lowest and highest iAUC (Figure 1B). Instead of a negative correlation, there was now a positive correlation with body weight (*r* = 0.488; *p* = 0.003). This means that when trying to correct the dose for body weight, the problem is reversed, thereby overdosing the heavy people and underdosing the less heavy people. Thus, although the principle of weight-corrected dosing seemed valid for BA, we simply replaced one problem by an equally large new problem, leading to zero progression toward homogeneity and individualized supplementation.

One possible explanation for the failure to improve homogeneity of supplement response could be that body weight is not the optimal scaling factor for dose calculation. Other body dimensions might more accurately reflect determinants of the pharmacokinetic response through for example GI tract dimension and therefore absorption surface, liver volume and thereby BA degradation capacity (8), kidney volume (15), blood volume (16), etc. However, other parameters, such as height, BMI, or RMR (Figure 2; Table 2) did not yield a better explained variance than body weight in the current study. Therefore, we believe that, when scaling is appropriate, body weight might still partially be a relevant scaling factor, as it is the easiest to translate and adopt to the population.

Since both the pharmacokinetic response of the FD and WRD suffered equally from an influence of body weight, yet in opposite directions, we now propose that supplement dosing should only partially be scaled to body weight. One approach would be to consider 50% of the dose to be given as a FD and 50% as a WRD, with a 70 kg person as a reference. Transferred to the example of a targeted 1,400 mg or 20 mg/kg BW BA, a 70 kg person would receive 700 mg as FD and 700 mg as 10 mg/kg body weight. Thus, with this scheme (700 mg as FD and 10 mg/kg BW as WRD), this would result in a 1,200 mg dose for a 50 kg person and 1,700 mg for a 100 kg person. An alternative approach would be to normalize the entire dose to only a portion of the body weight. When comparing anthropometrically diverse populations, scaling maximal oxygen uptake to body weight is usually done by dividing VO2 by 2/3rd of the lean body mass (17). The latter is less likely to be easily adoptable because it requires lean body mass determination and calculation beyond the general public's abilities. It is important to consider that, the dose-scaling strategies presented here can only be used as a suggestion since these were not tested in this study.

Another interesting observation was that the iAUC was non-lineary related to the dose (Figure 4). This implies that especially with higher dosage, a certain change of dose will not induce the same effect on the iAUC. In the former example this means that when a 100 kg person ingests a WRD of 20 mg/kg BW (2,000 mg) or a partially FD and WRD of 700 + 10 mg/kg BW (1,700 mg) this would result in a 87 and 40% increase of iAUC while the dose only increased 43 and 21%, respectively, compared to a FD of 1,400 mg. The fact that the dose is non-linearly related to iAUC adds an extra layer of complexity in future research investigating different dosing strategies.

In the current experiment, a quite diverse sample was included consisting of men and women, from different ethnicities and with different anthropometric characteristics. The current sample somewhat resembles the population toward which results should be generalized, although our sample did not include people above 105 kg and 1m94, which is a limitation. Current standard practice is to supplement athletes with a fixed dose. In this sample, the fixed dose of 1,400 mg resembled a relative dose of 13 and 30 mg/kg BW for the heaviest and lightest subjects respectively. A WRD of 30 mg/kg BW is already quite high when considering the pioneering study reported significant side-effects, although not recorded as unpleasant, at a dose of 20 mg/kg BW (3). On the other hand, including more heavy subjects would most likely have resulted in an even smaller physiological response when ingesting the fixed dose (< 13 mg/kg BW). In future research, it is the scientific community's responsibility to test and formulate recommendations for all athletes, also those with more extreme anthropometric characteristics.

Although other supplements are characterized by a different metabolism, similar dose related considerations have not yet been made for the other evidence-based performance supplements. Within each supplement there exists a more or less standard practice, with bicarbonate and caffeine being administered in WRD and creatine and nitrate mostly used in FD. Interestingly, it seems that the adopted dosing strategy is primarily based on the pioneering study. Jones et al. (18) were the first to investigate bicarbonate as an ergogenic supplement and used a WRD (0.3 g/kg), whereafter the community continued to do so. Creatine and nitrate are mostly used in FD in accordance with the pioneers who supplemented a FD of 5 g/dose, 4–6 times a day (3), and 500 ml/day beetroot juice (NO^3−^:5.5 mmol/day) (19) to improve exercise, respectively. In contrast, pioneering researchers used caffeine in a FD of 330 mg (20), but the scientific community shifted toward the use of WRD. One might suggest that, at this point, WRD is mostly used in ergogenic aids that are taken acutely and whereby overdosing might cause ergolytic effects. In contrast, convenient FD is used when supplements are chronically administered and when acute side effects are not likely to affect performance. In any case, there is at this point no scientific justification on why current dosing strategies are used in relation to optimization of personalized responses.

In the present study, a FD and WRD of 1,400 mg and 10 mg/kg BW were used, respectively. The FD of 1,400 mg corresponded to a relative dose of 13.40–30.43 mg/kg BW, whereas the WRD matched 460 mg up to 1,045 mg. These doses and the corresponding physiological response (iAUC) are different, making direct comparison between two dosing strategies used in this investigation impossible. We acknowledge this difference in absolute dose between de FD and WRD conditions as a limitation to the current study. Nonetheless, we deem the conclusions of the current data as valid and valuable.

In summary, the current study showed that—at least for the example of BA—neither FD nor WRD is adequate toward personalized nutrition, since in both cases the observed variation was equally high and partially explained and correlated to anthropometrics. This underscores the importance to better address the relationship between different doses of nutritional supplements and the physiological responses they elicit in an anthropometric diverse sample. Future studies will need to test more advanced dose-scaling strategies, where after it will be possible to provide scientifically based recommendations for provoking more homogenous physiological responses in athletes.

## Author contributions

JS, IE, and WD conceived and designed the experiment. JS, FL, and IE performed the experiment. JS, IE, and WD analyzed and interpreted the experiment. JS, IE, FL, and WD wrote and approved the final version of the manuscript.

### Conflict of interest statement

The authors declare that the research was conducted in the absence of any commercial or financial relationships that could be construed as a potential conflict of interest. The reviewer ED and handling Editor declared their shared affiliation.

## Footnotes

^1^Australian Sports Commission Group A sports supplement AIS. Available online at: https://www.ausport.gov.au/ais/nutrition/supplements/groupa (Accessed March 20, 2018).
